# Supplementary material for: Comparing and linking machine learning and semi-mechanistic models for the predictability of endemic measles dynamics
Source: PLoS Comput Biol. 2022 Sep 8;18(9):e1010251. doi: 10.1371/journal.pcbi.1010251 (PMC9455846; doi:10.1371/journal.pcbi.1010251)
Supplement: S2 Fig — (PDF) [file pcbi.1010251.s002.pdf]

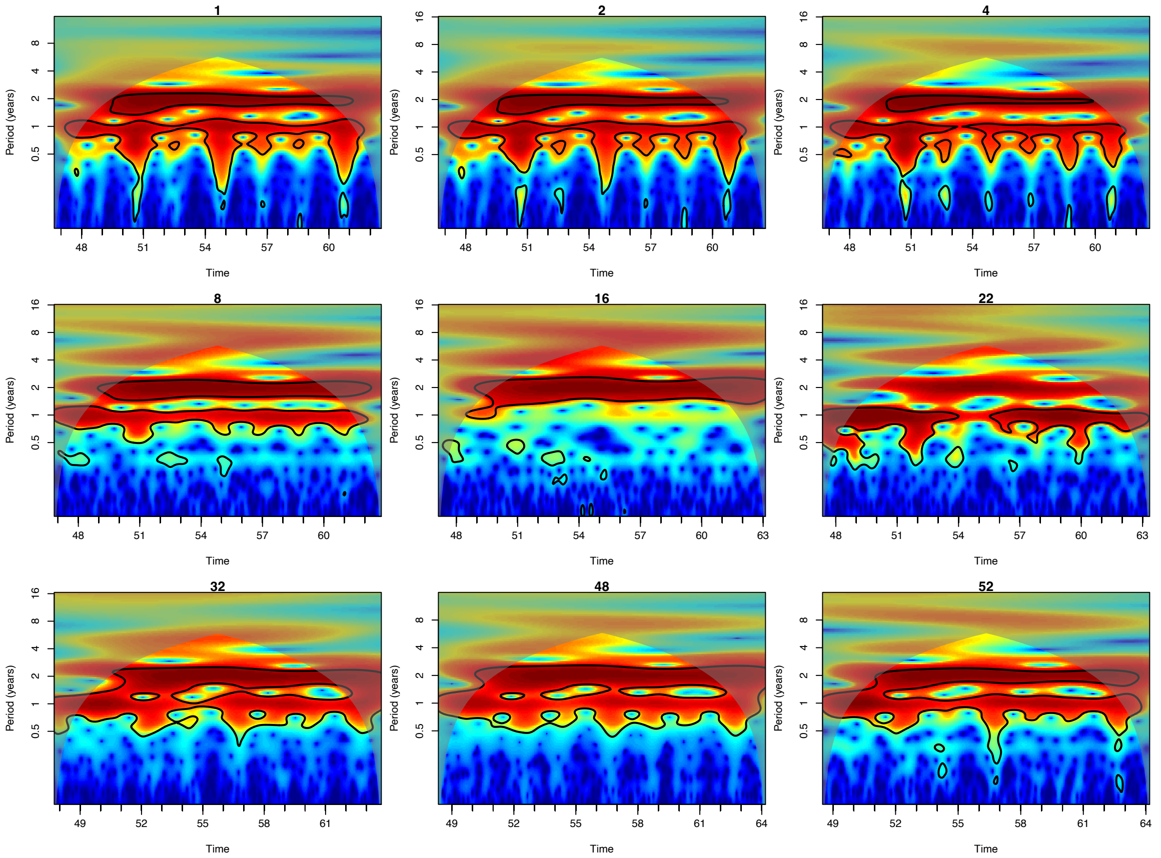

Fig. S2: Power spectra of the out of sample predictions for measles in London using the LASSO model (without using birth data in the LASSO model).
