# Supplementary material for: Comparing and linking machine learning and semi-mechanistic models for the predictability of endemic measles dynamics
Source: PLoS Comput Biol. 2022 Sep 8;18(9):e1010251. doi: 10.1371/journal.pcbi.1010251 (PMC9455846; doi:10.1371/journal.pcbi.1010251)
Supplement: S3 Fig — (PDF) [file pcbi.1010251.s003.pdf]

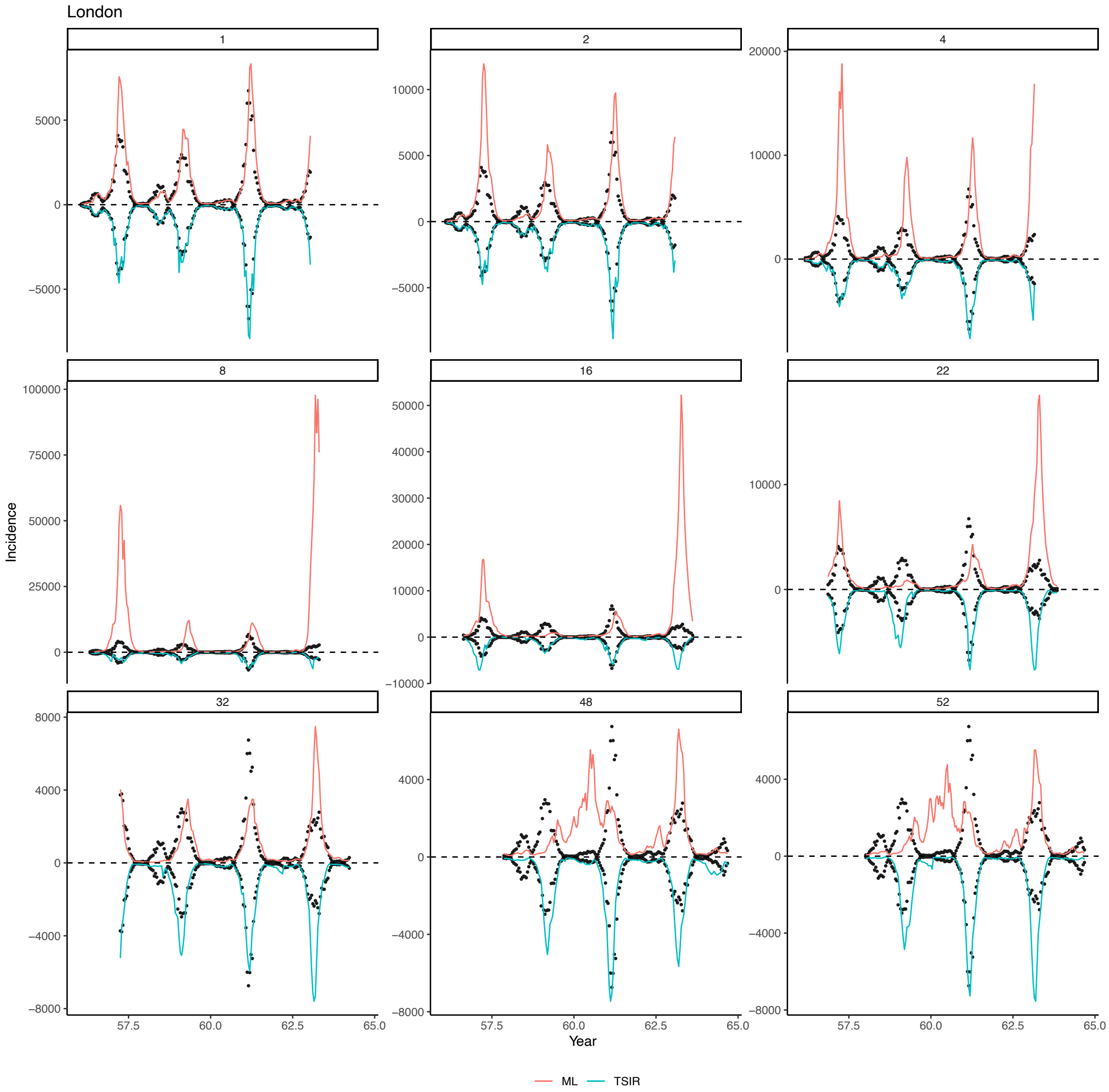

Fig. S3: A subset of 1 to 52th-biweek ahead *out-of-sample* (i.e. period excluding data in the training set) predictions from our LASSO model and the TSIR model, for pre-vaccination measles epidemics in London from 1944-64. Data between 1944-51 (*from London only*) are used to train the LASSO models.
