# Supplementary material for: Comparing and linking machine learning and semi-mechanistic models for the predictability of endemic measles dynamics
Source: PLoS Comput Biol. 2022 Sep 8;18(9):e1010251. doi: 10.1371/journal.pcbi.1010251 (PMC9455846; doi:10.1371/journal.pcbi.1010251)
Supplement: S4 Fig — (PDF) [file pcbi.1010251.s004.pdf]

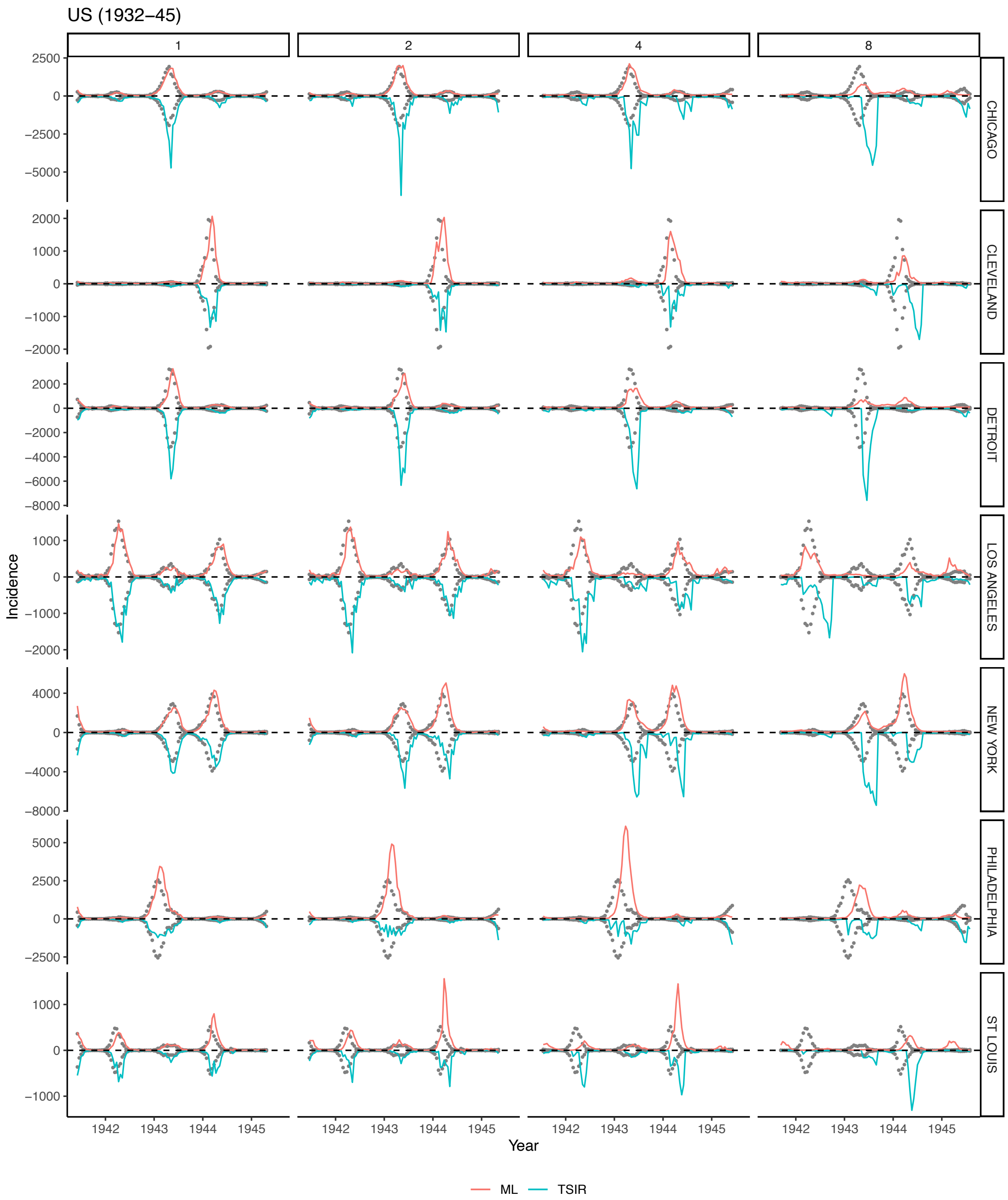

Fig. S4: A subset of 1 to 8th-biweek ahead out-of-sample predictions from our LASSO model and the TSIR model, for measles epidemics in 7 major cities in US from 1932-45. Data between 1932-40 are used to train the LASSO models.
