# Supplementary material for: Comparing and linking machine learning and semi-mechanistic models for the predictability of endemic measles dynamics
Source: PLoS Comput Biol. 2022 Sep 8;18(9):e1010251. doi: 10.1371/journal.pcbi.1010251 (PMC9455846; doi:10.1371/journal.pcbi.1010251)
Supplement: S5 Fig — (PDF) [file pcbi.1010251.s005.pdf]

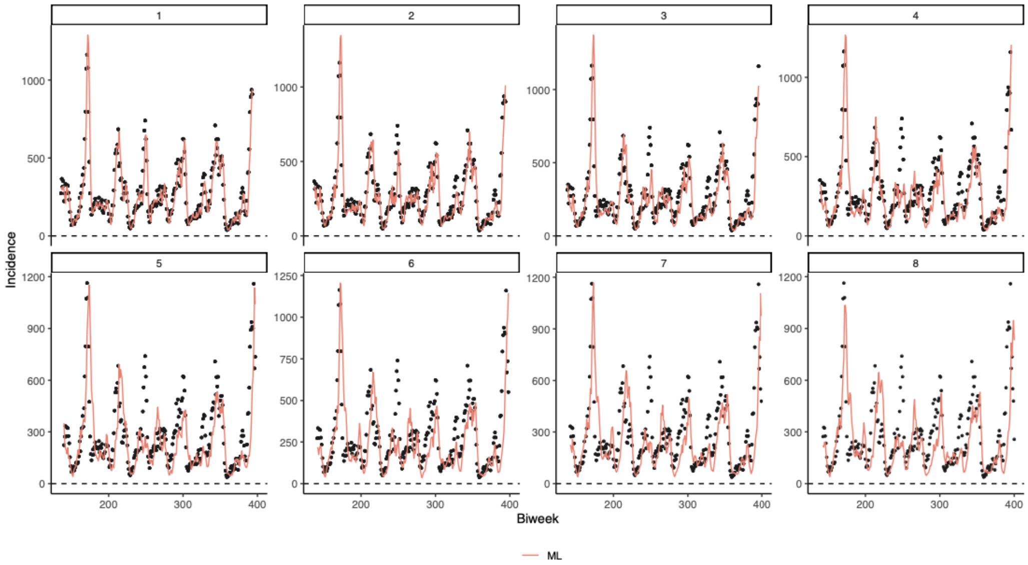

Fig. S5: Simulation studies. Our LASSO model is fitted to epidemics generated from a TSIR model. Specifically, local dynamics are simulated from the estimated TSIR model using the pre-vaccination E&W dataset. Using first half of the data for training, our LASSO model can reasonably well predict the outbreak trajectory.
